# Predictors of partnership and sexual satisfaction and dyadic effects in couples affected by endometriosis and infertility

**DOI:** 10.1007/s00404-024-07516-z

**Published:** 2024-05-06

**Authors:** Deborah van Eickels, Maren Schick, Ariane Germeyer, Sabine Rösner, Thomas Strowitzki, Tewes Wischmann, Beate Ditzen

**Affiliations:** 1 Institute of Medical Psychology, University Hospital Heidelberg, Heidelberg, Germany; 2 Department of Gynecological Endocrinology and Fertility Disorders, Heidelberg University Women’s Hospital, Heidelberg, Germany

**Keywords:** Endometriosis, Infertility, APIM, Sexuality, Partnership

## Abstract

**Purpose:**

Endometriosis and infertility are associated with impaired partnership and sexuality of the patients, but also of their male partners. Also, endometriosis is one of the most common causes of infertility, resulting in a large overlap of both pathologies. The aim of this study was to determine the association of different predictors of partnership and sexual satisfaction and dyadic effects in couples with endometriosis and infertility.

**Methods:**

A cross-sectional study was conducted with n = 62 women with endometriosis and n = 46 partners, including a total of n = 44 couples, some of whom were affected by infertility. The questionnaire included items on partnership, sexuality, depression, social support, and desire for a child. Multiple linear regression and the actor-partner-interdependence-model were used for analysis.

**Results:**

Significant dyadic effects only occurred in couples with both endometriosis and infertility. Depression showed a significant negative actor effect in men for partnership satisfaction and a negative actor and partner effect in women for sexuality satisfaction (p < .05). For women, social support showed a significant positive actor effect for partnership satisfaction (p < .05), age showed a significant actor and partner effect for sexuality satisfaction (p < .05).

**Conclusion:**

The results show a significant association of endometriosis and infertility with partnership and sexuality satisfaction. Infertility could be a decisive factor. However, the large overlapping of both endometriosis und infertility in many couples support the importance of further studies to differentiate between the both effects.

**Trial registration:**

German Clinical Trials Register DRKS00014362 on the 29.03.2018.

## What does this study add to the clinical work

**Table:** 

Studies on the effects of endometriosis and infertility have so far mainly taken into account only one of the pathologies and the perspective of affected women, while studies on the effects on couples are lacking. This study shows the need to consider not only affected women but also their male partners in their burden in clinical work and also to conduct further studies to differentiate between the effects of infertility and endometriosis and their interrelationships.	

## Introduction

Endometriosis affects 5–10% of women of childbearing age, with prevalence ranging from 2–11% in asymptomatic women to 5–50% in infertile women and has also been described as a contributing cause of infertility [1–3]. Regarding the German IVF Register, endometriosis was documented in 22.9% of all women undergoing infertility treatment in Germany in 2020, indicating both groups overlapping in large part [4]. Worldwide 8–12% of all couples suffer from infertility [5].

Whilst endometriosis can cause physical symptoms such as pain and dyspareunia previous studies also showed a connection of endometriosis with psychological stress in form of depression, anxiety, and stress. All these symptoms lead to an influence on the social and work life of the women as well as on their partnerships and sexual life [6–9]. In addition to the affected women, male partners also experience psychological stress due to the endometriosis affecting the woman as well as the partnership and their sexual life [10]. Symptoms reported by male partners comprise feeling helpless, frustration, worry, anger and stress [11]. Social support is reported rarely but is evaluated as positive when received [12]. Regarding the connection between endometriosis and sexuality, for women symptoms such as dyspareunia, sexual dysfunction and lower sexual satisfaction are consistently described in several studies [13–16]. For male partners, only few studies exist about the influence of endometriosis on sexuality, intimacy and partnership [10, 12, 17, 18]. Male partners do not seem to suffer consistently from sexual dysfunction but do report an influence on their sexual satisfaction in terms of a lower coital frequency due to the women’s pain while wishing for a higher frequency of sexual intercourse [13].

Infertility is also often related to psychological burdens such as depression, anxiety, stress and guilt in women but also in men of affected couples [19–22]. Sexual dysfunction and lower sexual satisfaction are reported in both men and women of infertile couples, with women showing a greater impairment [19, 20, 23–26].

Relationship satisfaction of infertile couples seems to be connected to the communication and way of coping with the infertility. Whilst some infertile women report that they have been left by their partners [27], most couples report a high relationship satisfaction despite the infertility [19, 20, 28]. This is linked to a “survival” of those relationships despite the infertility and sometimes even a stronger connection and better communication [20].

Since existing studies mostly focus only on endometriosis or only on infertility and studies investigating both women with endometriosis and their male partners are still lacking, the aim of this study was to identify predictors of sexual satisfaction (SS) and partnership satisfaction (PS) in couples with endometriosis and infertility. We aimed to investigate how these variables are related to each other, while taking into account the infertility factor.

## Materials and methods

### Setting and questionnaires

Between September 2016 and August 2018, all women who underwent laparoscopic surgery for infertility or dysmenorrhea at the Department of Gynecological Endocrinology at Heidelberg University Hospital—and in whom endometriosis could be confirmed—were informed about the study. All women and their male partners received further information about the study in writing, an informed consent form, and (if they consented) were each asked to complete a set of questionnaires (see below). The study pursued two objectives. Firstly, the effects of endometriosis-associated pain, and secondly, predictors of and the impact of infertility on partnership and sexuality satisfaction in couples with endometriosis. The results on the impact of endometriosis-associated pain in couples are reported elsewhere [29] (an extended multicentre sample was examined in this publication).

The data analysed included the results of the laparoscopic surgery and the patient files from the hospital as well as the handed-out questionnaires, including up to 88 items for female and 72 items for male participants.

Partnership satisfaction and social support were surveyed by questions from the Swiss Household Panel [30]. We used a visual analogue Scale (VAS) with a range from 0 (“not at all”) to 10 (“very much”). Two questions referred to the support and understanding from the social environment like relatives, friends, and colleagues. Both questions were summarized to the variable “social support”. For partnership satisfaction in total 4 questions were used, which addressed support, understanding, intimacy and happiness within the partnership. All 4 questions were summarized to the variable “partnership satisfaction”.

Regarding sexuality in total 3 questions were assessed. Sexual satisfaction was measured by using a VAS with a range from 0 (“very unsatisfied”) to 10 (“very satisfied”). Next to that frequency of sexual intercourse and for the women also the frequency of pain during sexual intercourse were asked. All the questions regarding sexuality were taken from the Female Sexual Function Index [31]. To determine the relative share of painful sexual intercourse we formed a ratio from the frequency of painful sexual intercourse and the frequency of sexual intercourse in total.

To assess depression, we used the German version of the “Depression, Anxiety and Stress Scale” [32]. The scale consists of 21 items regarding depression, anxiety, and stress, using a 4-point Likert-scale from 0 (“never”) to 3 (“almost always”). For each variable 7 items are summarized. Items for depression include questions about listlessness, mood, and joylessness. For depression a Cronbach’s Alpha = 0.91 was calculated by the authors of the questionnaire, showing a high internal consistency of the scale.

Sociodemographic data included age, level of education, current profession, marital status, number of children, duration of  desire for a child, partnership duration, subjective cause of infertility and kind of infertility treatment, as well as status, duration, and treatment of endometriosis.

### Data analysis

Regarding the status of endometriosis and desire to have children, data from women with endometriosis and their male partners were used and this group was further subdivided according to an actual existing desire for a child (yes/no).

Testing was done by using Mann-Whitney-U-Tests and chi-squared-tests (depending on the variable’s quality) for between-group comparisons between the men and women with and without infertility. To determine the predictors for sexual and partnership satisfaction we performed multiple linear regressions with the whole sample.

To evaluate how the predictors for sexual and partnership satisfaction interact within a couple and whether they show a mutual effect between men and women we used the Actor-Partner-Interdependence-Model (APIM) by David A. Kenny. This model assumes that one person’s behaviour or a predictor not only shows an effect on him- or herself (actor effect) but also on his/her partner (partner effect). In our APIM, we used the predictors identified in the multiple linear regression as the independent variable and sexual and partnership satisfaction as the dependent variable. The web program APIM_MM (Actor Partner Interdependence Model with Multilevel Modeling) for distinguishable dyads by David A. Kenny (http://davidakenny.net/DyadR/DyadRweb.htm) was used to calculate the APIM. This web program also calculates effect sizes (ES) to estimate the clinical relevance of the APIM effects.

## Results

### Sociodemographic data

380 questionnaires were handed out to the patients and their partners; a total of 161 participants completed the questionnaires (response rate: 42.37%). After excluding incomplete data sets and patients without endometriosis, n = 62 women with endometriosis and n = 46 male partners were included in the analysis. In total there were n = 44 heterosexual couples among the participants.

Descriptive statistics for men and women are shown in Tables 1 and 2.

**Table 1:** Descriptive statistics women

	Total (n = 62)	Endo + /Inf−* (n = 15)	Endo + /Inf + ** (n = 47)	p-value
	Mean (SD) / N (%)	Mean (SD) / N (%)	Mean (SD) / N (%)	
Sociodemographic				
Age	31.00 (5.30)	27.87 (7.20)	32.00 (4.16)	**0.024**^**a**^
Partnership duration in years	7.15 (4.37)	4.62 (3.81)	7.64 (4.34)	**0.032**^**a**^
Child present already	10 (16.2)	2 (13.4)	8 (16.0)	1.000^c^
Education				0.118^b^
No degree	–	–	–	
Secondary school	37 (59.7)	11 (73.3)	26 (55.3)	
University	24 (38.7)	3 (20.0)	21 (44.7)	
Endometriosis				
New diagnosis	7 (11.3)	1 (6.7)	6 (12.8)	1.000^c^
Infertility	47 (75.8)			
Duration of desire for a child (years)			2.81 (2.10)	
Psychosocial				
Depression	4.61 (5.24)	6.53 (5.22)	3.98 (5.14)	**0.019**^**a**^
Social support	7.59 (2.41)	7.65 (2.90)	7.57 (2.27)	0.469^a^
Partnership satisfaction	8.69 (1.92)	8.64 (1.98)	8.70 (1.93)	0.850^a^
Sexuality				
Sexual satisfaction	7.22 (3.34)	6.00 (3.64)	7.48 (3.25)	0.204^a^
Frequence of sex. intercourse (last 4 weeks)				0.090^c^
Never	9 (14.5)	5 (33.3)	4 (8.5)	
Once/Month	9 (14.5)	3 (20.0)	6 (12.8)	
Once/Week	18 (29.0)	2 (13.3)	16 (34.0)	
Several times/Week	19 (30.6)	4 (26.7)	15 (31.9)	

*Endo + /Inf− = subgroup with endometriosis, no infertility

**Endo + /Inf +  = subgroup with endometriosis und with infertility

^a^Mann-Whitney-U-Test

^b^Pearson’s chi-squared-test

^c^Fisher’s exact test

**Table 2:** Descriptive statistics men

	Total (n = 46)	Endo + /Inf-* (n = 9)	Endo + /Inf + ** (n = 37)	p-value
	Mean (SD) / N (%)	Mean (SD) / N (%)	Mean (SD) / N (%)	
Sociodemographic				
Age	35.54 (6.26)	33.44 (9.45)	36.05 (5.27)	0.397^a^
Partnership duration in years	7.16 (4.41)	5.36 (4.03)	7.51 (4.45)	0.193^a^
Child present already	9 (19.5)	4 (44.4)	5 (13.5)	**0.039**^c^
Education				0.243^c^
No degree	–	–	–	
Secondary school	23 (50.0)	6 (66.7)	17 (45.9)	
University	22 (47.8)	2 (22.2)	20 (54.1)	
Endometriosis				
New diagnosis	5 (10.9)	1 (11.1)	4 (10.8)	1.000^c^
Infertility	37 (80.4)			
Duration of desire for a child (years)			2.62 (1.76)	
Psychosocial				
Depression	2.11 (3.15)	4.00 (5.66)	1.70 (2.22)	0.327^a^
Social support	6.50 (2.69)	6.11 (3.19)	6.59 (2.59)	0.643^a^
Partnership satisfaction	9.14 (1.18)	7.90 (2.13)	9.44 (0.51)	0.051^a^
Sexuality				
Sexual satisfaction	7.64 (2.64)	6.44 (4.02)	7.94 (2.13)	0.492^a^
Frequence of sexual intercourse (last 4 weeks)				0.433^c^
Never	8 (17.4)	3 (33.3)	5 (13.5)	
Once/Month	7 (15.2)	2 (22.2)	5 (13.5)	
Once/Week	13 (28.3)	1 (11.1)	12 (32.4)	
Several times/Week	13 (28.3)	3 (33.3)	10 (27.0)	

*Endo + /Inf− = subgroup with endometriosis, no infertility

**Endo + /Inf +  = subgroup with endometriosis und with infertility

^a^Mann-Whitney-U-Test

^b^Pearson’s chi-squared-test

^c^Fisher’s exact test

Regarding the presence of infertility there were significant differences between the two groups in several variables. Mean age and duration of the partnership were significantly higher in infertile women in comparison to women without infertility (p = 0.024 for age and p = 0.032 for duration of partnership), whereas level of depression was significantly lower (p = 0.019). Men without a desire for a child were significantly more likely to be a father already than men with a desire for a child (p = 0.039). Regarding the other variables there were no significant differences.

### Predictors of partnership and sexual satisfaction: multiple linear regression for all individuals

In the multiple linear regression for PS for all women with endometriosis and male partners of women with endometriosis with the predictors sex, age, duration of partnership, depression, social support, and desire for a child a variance explanation of 0.12 (corrected R^2^) was reached. Depression was correlated with lower PS (p = 0.034). The positive correlation between social support and PS was almost significant (p = 0.051) (Table 3).

**Table 3:** Multiple linear regression for partnership satisfaction with endometriosis

	Not standardized coefficients		Standardized coefficients	T	Sig	95,0% CI for B^a^	
	Regression coefficient B	Standard error^a^	Beta			Upper	Lower
(Intercept)	0.118	0.210		0.536	0.593	− 0.286	0.485
Gender	0.143	0.099	0.139	1.273	0.207	− 0.048	0.341
Age	0.078	0.058	0.135	1.192	0.237	− 0.021	0.185
Partnership duration	0.040	0.048	0.078	0.758	0.450	− 0.049	0.114
Depression*	− 0.128	0.088	− 0.228	− 2.155	**0.034**	− 0.326	− 0.001
Social support*	0.105	0.051	0.210	1.976	0.051	0.007	0.224
Infertility	− 0.111	0.153	− 0.078	− 0.740	0.461	− 0.401	0.199

Model-Test significant (p = 0.01), n = 94

^a^Bootstrapping result based on 1000 replicates

*Significance confirmed in Bootstrapping

In the multiple linear regression for SS for all women with endometriosis and male partners of women with endometriosis with the predictors sex, age, duration of partnership, depression, and frequency of sexual intercourse and desire for a child a variance explanation of 0.37 (corrected R^2^) was reached. Depression was correlated with lower SS (p = 0.004). The frequency of sexual intercourse was correlated with higher SS (p < 0.001) (Table 4).

**Table 4:** Multiple linear regression for sexual satisfaction with endometriosis

	Not standardized coefficients		Standardized coefficients	T	Sig	95,0% CI for B^a^	
	Regression coefficient B	Standard error^a^	Beta			Upper	Lower
(Intercept)	− 0.312	0.377		− 0.792	0.431	− 1.031	0.489
Gender	− 0.035	0.196	− 0.016	− 0.172	0.864	− 0.433	0.293
Age	0.088	0.165	0.064	0.662	0.510	− 0.198	0.370
Partnership duration	− 0.032	0.094	− 0.030	− 0.323	0.748	− 0.230	0.180
Depression *	− 0.290	0.148	− 0.251	− 2.679	**0.009**	− 0.577	− 0.040
Frequency sexual Intercourse*	0.645	0.138	0.517	5.588	0.**000**	0.393	0.900
Infertility	0.192	0.268	0.068	0.720	0.474	− 0.327	0.718

Model-Test significant (p < 0.001), n = 83

^a^Bootstrapping result based on 1000 replicates

*Significance confirmed in Bootstrapping

### Predictors of partnership and sexual satisfaction: dyadic analysis using the APIM

The desire for a child had a significant effect in women with endometriosis and their male partners as a covariate in the APIM analysis of partnership satisfaction. Depression and social support did not show any significant actor or partner effects in the APIM analysis for PS. Using only the data of couples affected by endometriosis and the desire for a child, depression showed a significant negative actor effect for male partners with a medium effect size (p = 0.021, r = −0.410). Social support showed a significant positive actor effect for women with a medium effect size (p = 0.046, r = 0.363).

**Fig. 1:**
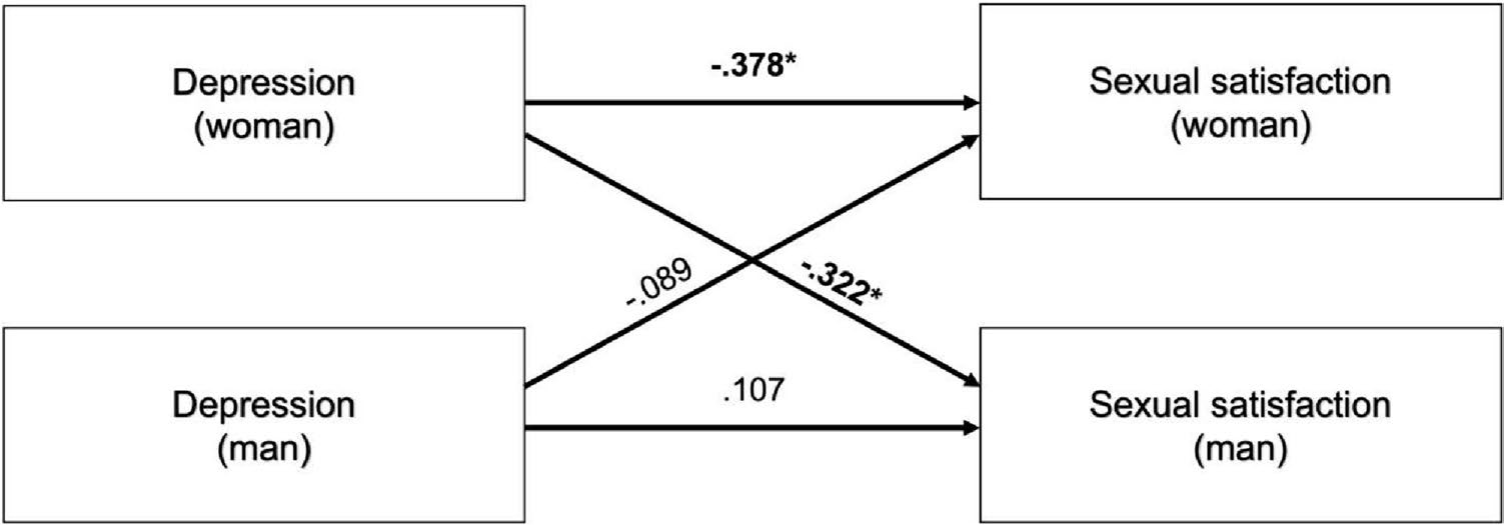
Actor- and partner-effects for depression on sexual satisfaction; Indication of effect size r; * = p < 0.05

The desire for a child also had a significant effect in women with endometriosis and their male partners as a covariate in the APIM analysis of sexual satisfaction. Depression did not show any significant actor or partner effects in the APIM analysis for SS. Using only the data of couples affected by endometriosis and the desire  for a child, depression showed a significant negative actor-effect for women with a medium effect size (p = 0.027, r = − 0.378) as well as a significant negative partner-effect (woman ⇒ man) with a medium effect size (p = 0.019, r = − 0.322) (Fig. 1). Age showed a significant positive actor-effect for women with a medium effect size (p = 0.044, r = 0.347) as well as a positive partner-effect (woman ⇒ man) with a medium effect size (p = 0.005, r = 0.451) (Fig. 2).

**Fi﻿g. 2:**
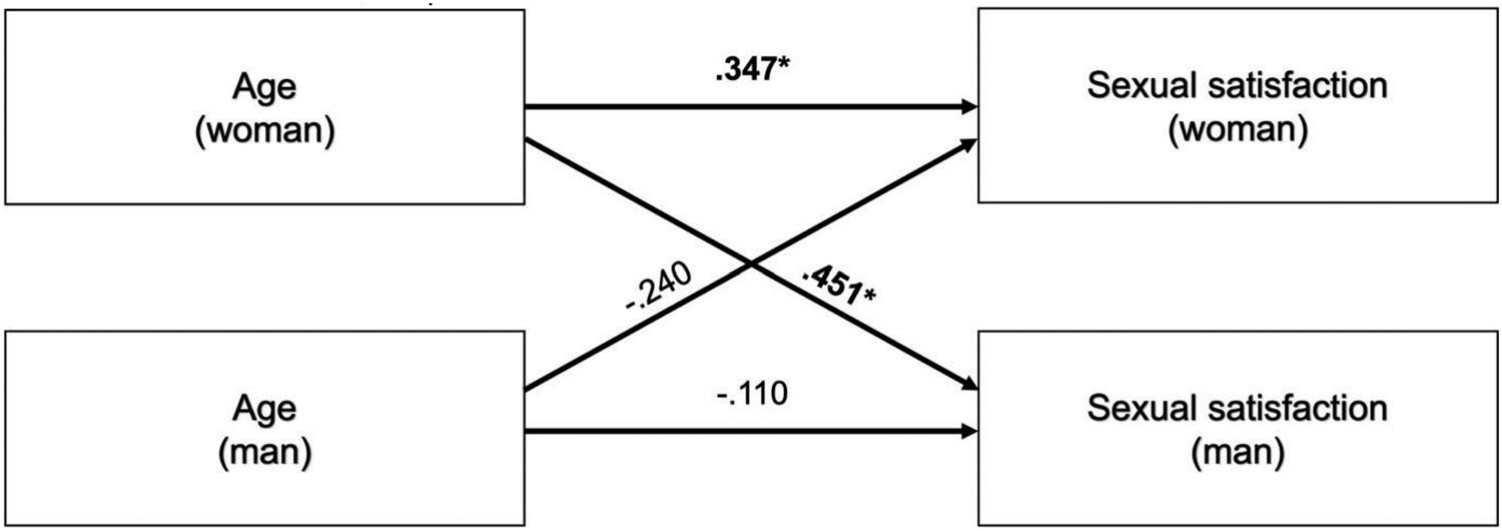
Actor- and partner-effects for age on sexual satisfaction; Indication of effect size r; * = p < 0.05

## Discussion

The results showed a significant correlation of depression, social support, pain, and desire for a child with PS and SS in couples affected by endometriosis. Also, the APIM showed significant differences between female and male partners.

### Depression and partnership satisfaction

The multiple linear regression showed a negative correlation between depression and PS. This result is in line with results of previous studies about women with endometriosis, which also showed a connection of endometriosis with depression and anxiety [6–9] and also a negative correlation of those two factors with PS [14, 15, 33]. A direct correlation between depression and PS in male partners of women with endometriosis has not been described yet. On the other hand, there are several studies showing an influence of endometriosis on the lifestyle and partnership of the male partners [10–12]. As a negative correlation between depression and partnership is described in both men and women of couples with infertility [34], a similar correlation for male partners of women with endometriosis is likely. In our APIM-Analysis only the couples with desire for a child showed significant effects, precisely there was a negative actor-effect of depression for the male partners. For women, neither an actor- nor a partner-effect could be found. An explanation might be that in our sample women with desire for a child showed significantly lower values for depression. However, the desire for a child was a significant covariate in the APIM analysis with all couples with endometriosis. This result might be explained by the described high partnership satisfaction of infertile couples in other studies [19, 20, 28].

### Social support and partnership satisfaction

Furthermore, the results of the multiple linear regression showed a positive correlation between social support and PS. This result is also in line with previous studies on women with endometriosis and their male partners [10, 12, 35], and this is also described for other chronic diseases [35]. In our APIM-Analysis again only the couples with desire for a child showed significant effects. An explanation might be that couples who also have an unfulfilled desire to conceive in addition to endometriosis receive more support from their social environment (or perceive it as such). The diagnosis of endometriosis could also become more understandable to outsiders in terms of its far-reaching effects. In our APIM-Analysis women showed a positive actor-effect, in previous studies a positive effect of social support on the PS was also described for male partners of women with endometriosis [12]. A similar finding regarding the difference between women and men in terms of social support was described for couples, in which one partner had cancer [36]. A meta-analysis about gender differences showed that women give (and therefore also receive) significantly more social support then men [37].

### Depression and sexual satisfaction

The multiple linear regression showed a negative correlation for depression and SS, which is in line with previous studies, showing a negative correlation of depression with SS in women with endometriosis [13–16]. Also, the negative correlation of depression and SS was described for women and men in previous studies and in a meta-analysis [38, 39]. Our APIM-analysis with the couples with a desire for a child showed negative actor- and partner-effects for depression on SS. The actor-effect might be traced back to the direct correlation of depression and SS, while the negative partner-effect might be explained by the interaction with the male partner, which might be affected by pain or depression.

### Frequency of sexual intercourse and sexual satisfaction

The positive correlation of the frequency of sexual intercourse and SS is an expected result. A higher frequency of sexual intercourse is probably connected to a higher satisfaction. Also, women with endometriosis and a high frequency of sexual intercourse might experience less pain symptoms and therefore less dyspareunia.

### Age and sexual satisfaction

The APIM-analysis for SS with the couples with desire to conceive showed a positive actor- and partner-effect for age in women. Previous studies did not describe an explicit effect of age on the SS in women in reproductive age. In one study, a higher age when getting married was described as a protective factor, but a higher age in total also as a risk factor for sexual dysfunction [40]. In our sample the positive effect of age might have several reasons. As a symptom reduction with higher age was described in patients with endometriosis, this might also have an influence on dyspareunia. Also, older women might have found a suitable therapy for their endometriosis. For example, in one study women described a higher quality of sexuality and a reduction of dyspareunia after a surgical therapy [41]. A longer partnership duration and better communication within the couple might have a positive effect on the sexual life and SS as well. A factor that always has to be kept in mind when doing surveys about sexuality is the social desirability in the response behavior [42].

### Strengths and limitations

This study shows several strengths but also limitations. One strength of the study is that next to endometriosis we also took the factor infertility into account and could show significant differences between couples with and without desire for a child. A further strength is the inclusion of male partners of the women with endometriosis, which enables a further understanding of the influence and effects of endometriosis on the life of male partners. The high number of couples allowed us to do pairwise analysis with the APIM.

However, regarding the factor infertility, we also see a limitation of this study. As we did not include couples without endometriosis and infertility or couples with infertility only, we did not have a control group, which would be necessary to have a more precise differentiation between the effects of endometriosis vs. infertility. As endometriosis is often only diagnosed in the process of an infertility treatment, it is probably difficult to find a sample with clearly separated groups. Another limitation is the low number of men and women in the several sub-groups, which did not allow us to perform subgroup analysis with a robust result. Also, we only had heterosexual couples in our study. Especially regarding the factors PS and SS studies with non-heterosexual couples would also be necessary to get a complete picture. Regarding the questionnaires, we only used extracts from the FSFI for the questions about sexuality. This might have reduced the informative value and accuracy of the data [42].

### Further implications

In summary, variables associated with endometriosis and infertility, such as depression and pain, were related to sexuality and partnership satisfaction in our study. This effect was particularly evident not only in affected women but also in male partners. In total this finding summarizes also results of previous studies which already described the effect of endometriosis on different parts of life for affected women and male partners. However, our results also showed significant differences regarding actor- and partner-effects in the dyadic analysis between couples with and without desire to conceive.

Regarding the large overlapping in both groups, this emphasizes the importance of taking the infertility into account in further studies on patients and couples with endometriosis. Especially to further differentiate the effects of endometriosis and infertility further studies are important in which both groups and control groups with and without endometriosis or infertility are included. Overall, studies on both endometriosis and infertility should always consider both factors independently as well as in their interdependence.

## Data Availability

The datasets used and/or analysed during the current study are available from the corresponding author on reasonable request.
